# NEIGHBORHOOD SOCIAL ENVIRONMENT AND PHYSICAL FUNCTION: EVIDENCE OF RACIAL AND ETHNIC DIFFERENCES

**DOI:** 10.1093/geroni/igz038.1034

**Published:** 2019-11-08

**Authors:** Roberto J Millar

**Affiliations:** University of Maryland, Baltimore County, Baltimore, Maryland, United States

## Abstract

Empirical and theoretical scholarship suggest that as individuals age and their physical, cognitive, and social needs change, their neighborhood environment becomes increasingly important to their health and well-being. Despite recent advances in this area of research, a number of critical gaps remain. Namely, few studies examine the associations between neighborhood social environments and performance-based physical function. Furthermore, racial and ethnic differences are widely understudied. The objectives of this study are (1) to examine the association between neighborhood social cohesion and physical disorder on physical function in older adults, and (2) to identify potential racial/ethnic differences in these associations. Data come from round five (collected in 2015) of the National Health and Aging Trends Study (NHATS; N=5,619). A series of adjusted linear regression models were used to predict performance-based physical function based on characteristics of the neighborhood social environment (i.e., cohesion, disorder). Results showed that only neighborhood physical disorder was statistically significantly associated with poorer physical function (p < 0.05). Similarly, when stratified by race/ethnicity, only neighborhood physical disorder was associated with poorer physical function in Whites (p < 0.05). There was no significant association for either neighborhood social environment characteristic and physical function for Black or Hispanic older adults. Racial and ethnic differences warrant closer investigation in studies of neighborhood effects on health. Community-level interventions, policy makers, and researchers should consider the interactions between minority membership and neighborhood social environments when addressing issues of health and physical function.

